# Guided imagery for treatment (GIFT): protocol of a pilot trial of guided imagery versus treatment as usual to address radiotherapy-related distress in head and neck cancer

**DOI:** 10.1186/s40814-022-01134-9

**Published:** 2022-09-05

**Authors:** Elissa Kolva, Sana D. Karam, Alaina L. Carr, Sydneyjane Roberts, Kathleen Torkko, Ryan Lanning, Emily Cox-Martin

**Affiliations:** 1 Division of Medical Oncology, Department of Medicine, School of Medicine, University of Colorado — Anschutz Medical Campus, MS 8117 12801 E. 17th Ave, Aurora, CO 80045 USA; 2 Department of Radiation Oncology, University of Colorado Denver — Anschutz Medical Campus, Aurora, CO USA; 3 Department of Psychology, University of Colorado Denver, Denver, USA; 4 Department of Pathology, University of Colorado Anschutz Medical Campus, Aurora, CO USA

**Keywords:** Guided imagery, RCT, Cancer, Oncology, Head and neck cancer, Radiotherapy (RT), Distress, Cancer-related distress

## Abstract

**Background:**

Cancers of the head and neck region are associated with high symptom burden and elevated levels of psychological distress. Radiotherapy (RT) is a common treatment for patients with head and neck cancer (HNC) that is associated with psychological distress related to the immobilizing nature of the treatment, frequency of treatment delivery, and side effects. Guided imagery is a relaxation technique that is beneficial in reducing psychological distress in patients with other cancer diagnoses but has not been studied in this patient population. The purpose of this study is to evaluate the feasibility and acceptability of a brief guided imagery intervention (guided imagery for treatment, GIFT) to reduce RT-related anxiety and depression in patients with HNC relative to treatment as usual (TAU).

**Methods:**

Patients with HNC planning to receive RT will be recruited to participate in a randomized controlled trial evaluating a brief, two-session guided imagery intervention (GIFT) relative to TAU alone. Primary aims include acceptability and feasibility evaluated through quantitative and qualitative methods. Measures of anxiety and depression, symptom burden, health-related quality of life, and anxiolytic medication use will be collected at baseline, during treatment, and at 1-month follow-up.

**Discussion:**

There are no published interventions of guided imagery for anxiety and depression in patients with HNC despite its efficacy in other populations of patients with cancer. This proposed project evaluates the feasibility and acceptability of an intervention that has the potential to reduce psychological distress in a vulnerable population. Additionally, we will preliminarily examine the impact of behavioral intervention on psychological distress and the use of anxiolytic medication, a novel area of study.

**Trial registration:**

Clinicaltrials.gov NCT03662698; registered on 9/6/2018.

**Supplementary Information:**

The online version contains supplementary material available at 10.1186/s40814-022-01134-9.

## Background

Patients with head and neck cancer (HNC) comprise a unique population due to their high risk for treatment failure and death [1, 2] and heavy symptom burden [3]. Most notably, they are at risk for facial disfigurement and functional changes in ability to speak, breathe, eat, and swallow [4–7]. Furthermore, patients with HNC report high levels of psychological distress and social isolation [8–12] compared to patients with other cancer diagnoses, with more than half of patients reporting persistent psychological distress [13]. Additionally, their risk for suicide is four times that of the general population [14]. Evidence shows that much of their distress is directly related to the treatments they undergo, including radiotherapy (RT [8];).

HNC is highly prevalent with approximately 650,000 cases diagnosed each year [15], and yet it is understudied in the field of behavioral medicine [7]. This is surprising given the high levels of psychological distress experienced by these patients and the high prevalence of smoking and heavy alcohol use [16] which can complicate adjustment to diagnosis and treatment [5]. Additionally, both substance use and psychological distress are predictors of patient survival [17], with lower survival rates for cancer patients who report symptoms of depression (71% versus 86%) [18].

Radiotherapy (RT), a standard treatment for HNC, is associated with high levels of psychological distress [8, 19]. RT involves daily treatments often for weeks at a time. Psychological distress increases over the course of RT, peaking at week 5 of treatment [8]. Treatment begins with a computed tomography “CT” simulation to determine accurate positioning while undergoing RT. During this simulation, the patient’s body is immobilized through the use of a face mask that will be used for each treatment. This face mask is bolted to the treatment table to restrict the patient’s movement for the duration of the therapy, which can cause treatment-related anxiety [20]. Prior to initiating RT, rates of clinically significant anxiety range from 20 to 47% in patients with HNC, depending on assessment measure [18, 21, 22]. Pretreatment depression tends to be lower than anxiety but increases over the course of treatment and persists post-RT. [21, 23, 24] For patients with HNC undergoing RT specifically, levels of depression have been associated with decreased overall survival [19]. It is critical that these difficulties be addressed given the direct relationships found between mental health and clinical outcomes in patients with HNC.

Guided imagery is a relaxation technique involving the visualization of images and is considered an adjuvant cancer therapy [25]. A systematic review of guided imagery in patients with heterogeneous cancer diagnoses found positive effects on depression, anxiety, and quality of life compared to patients in a control group [25]. It has been found to enhance comfort and quality of life and reduce anxiety and fatigue in women undergoing RT for breast cancer [26]. Although it has been useful in reducing anxiety in other cancers and with other treatment modalities, there are no studies to date specifically examining guided imagery for patients with HNC undergoing RT.

The primary objective of this study is to evaluate the feasibility and acceptability of a manualized brief guided imagery intervention, guided imagery for treatment (GIFT) plus treatment as usual (TAU), to reduce RT-related anxiety and depression in patients with HNC relative to TAU alone. This study will provide information about the appropriateness of the intervention with this population and feasibility of implementation in a clinical setting. The secondary objective for this study is to evaluate the impact of the GIFT intervention on symptoms of anxiety and depression in patients with HNC comparted to TAU.

## Methods

### Setting

This study will be conducted at the University of Colorado Cancer Center, a NCCN-designated Comprehensive Cancer Center in the Mountain West. Participants will be recruited from the Department of Radiation Oncology. It was registered with Clinicaltrials.gov NCT03662698. Ethical approval was obtained from the Colorado Multiple Institutional Review Board (COMIRB).

### Study design

This paper reports the protocol (COMIRB no. 18-1100 v. 7.22.20) for a longitudinal two-arm randomized control trial (RCT) comparing the GIFT intervention for treatment of RT-related distress in patients with HNC undergoing RT to TAU. A 1:1 allocation ratio will be used for randomization. Participants may be randomized to the GIFT condition, which includes both TAU and participation in the GIFT intervention or TAU alone. The primary outcome of this pilot study is evaluation of the feasibility and acceptability of the GIFT intervention. This will be accomplished through evaluation of participant recruitment, completion of study measures, and retention. Qualitative data will provide insight on the acceptability of the study procedures and the intervention process. A secondary outcome is the preliminary efficacy of the GIFT intervention to impact self-reported anxiety, depression, health-related quality of life, and symptom burden. Additionally, the study will track anxiolytic medication use in patients with HNC as an exploratory outcome. See Table 1 for a schematic outlining the complete timeline and list of activities for participants in this trial.

**Table 1:** GIFT protocol schedule of assessments and procedures

	Screening and enrollment	Prior to RT	During radiation therapy (RT)^a^							Post-RT
	Baseline	CT simulation visit	Week 1	Week 2	Week 3	Week 4	Week 5	Week 6	Week 7	Week 12
Enrollment										
Consent	X									
Eligibility^b^	X									
Randomization^c^	X									
Intervention										
CT simulation		X								
In-person or virtual GI session		X	X							
Assessment										
Demographics	X									
Patient self-administered GI use^d^			X	X	X	X	X	X	X	
Anxiety HADS-A	X		X		X				X	X
Depression HADS-D	X		X		X				X	X
Symptoms MSAS-SF	X				X				X	X
Health-related QOL FACT-HN	X				X				X	X
Intervention use TLFB-I^d^		X	X	X	X	X	X	X	X	
Anxiolytic use TLFB-A		X	X	X	X	X	X	X	X	
Qualitative interview										
Research assistant contact for F/U^e^										X
Qualitative interview^f^										X

^a^Patients may complete the assessments for weeks 1, 3, 7, and 12, listed above any time during the specified week (7 days) of treatment, with day 1 being defined as the first day of radiation treatment. TLFB measures may be completed at any time

^b^determined by chart review and confirmation of eligibility between research study personnel and treating physician

^c^upon completion of baseline measures

^d^guided imagery (intervention) participants only

^e^following completion of 12-week study measures, in person or on the phone; treatment as usual participants will be provided with an MP3 player containing the guided imagery audio files either in person or by mail. Intervention participants will be offered the opportunity to participate in a 30-min qualitative interview assessing the acceptability and feasibility of the intervention

^f^the qualitative interview may be conducted via phone or in person following completion of the week 12 study assessments

### Participants

We will recruit patients at the University of Colorado Cancer Center who are initiating RT for a HNC diagnosis. This will include patients with diagnoses that include the oral cavity, pharynx, larynx, paranasal sinuses and nasal cavity, and salivary glands. Participants will be initially screened for eligibility via chart review. Eligible participants will be approached for consent following their initial visit with the Head and Neck Multidisciplinary Clinic. HNC patients are eligible to participate if they are as follows:

1. Have a pathology confirming HNC diagnosis
2. Are initiating RT at the University of Colorado Cancer Center
3. Have psychiatric stability as determined by chart review and clinician assessment. Patients with unmanaged serious mental illness or cognitive impairment are ineligible.
4. Are able to speak, read, and understand English

A study research assistant will communicate eligibility to the patient’s treating radiation oncologist and will approach eligible participants in person, assess interest in study participation, and obtain informed consent. Following the informed consent process, the research staff member will utilize the randomization function of REDCap data management program to randomize them to either the GIFT or TAU condition. This allocation sequence is locked to all research staff, including the PI. In REDCap, once group assignment has occurred, the field becomes read only and cannot be changed. Blinding will not be utilized in this study given the nature of the intervention and study design.

### Outcome measures

#### Feasibility

The primary aim of the study is the acceptability of the GIFT intervention and the feasibility of recruiting for and delivering the intervention in the radiation oncology setting. Feasibility will be demonstrated by the number of eligible participants referred from the radiation oncology clinic and enrolled in the study. Participant completion of intervention sessions, study measures and procedures, and study retention will provide additional feasibility data.

#### Acceptability

Acceptability will be evaluated by participant-reported use of the guided imagery skills taught in the GIFT intervention assessed through timeline follow-back method (TLFB [27];). Participants will be asked to complete a weekly TLFB measure via a HIPPA secure REDCap link to ascertain a retrospective, calendar-based, daily estimate of use of the guided imagery skills. Participants who do not return their TLFB data will be contacted by study staff who will administer the recall via telephone. The TLFB is a reliable measure of patient-reported substance use (i.e., cigarettes, cannabis, and alcohol [27];).

GIFT intervention participants will be invited to complete a qualitative interview that will further assess the acceptability of the intervention. We expect that themes will emerge from the qualitative data that will indicate general acceptance and usefulness of guided imagery. The interviews will be conducted by a study team member who will be appropriately trained in qualitative methodology. Interviews will be conducted using a semi-structured interview protocol [28], which will be given either in person or over the telephone. The interview will be recorded, transcribed, and analyzed. Interviews will last approximately 30 min. Participants who participate in the qualitative interviews will receive at US $25 gift card in compensation for their time.

#### Secondary outcomes

The study will provide support for preliminary efficacy of the GIFT intervention. It will provide data on depression, anxiety, health-related quality of life, and symptom burden for patients with HNC undergoing RT. All participants will complete assessments at baseline, following initiation of RT (week 1), approximately halfway through RT (week 4), following the end of RT (week 7), and 1 month following completion of RT (week 12, see Table 2). These self-report data will be collected via email link connected to a secure REDCap database (see Table 1 for study data time points). These assessments can also be conducted in clinic via electronic tablet. Participants who do not complete their surveys will be contacted by research staff who will administer the questionnaires over the telephone.

**Table 2:** Content of the GIFT interventions

Session	Session goals	Homework
1 — conducted the week of CT simulation	• Introduce intervention and confidentiality • Build rapport through sharing cancer story • Identify impact of stress on the body • Introduce guided imagery practice • Orient to mp3 player use	• Self-administration of guided imagery vignette • Begin tracking intervention use and benzodiazepine use
2 — conducted during the first week of RT	• Review guided imagery use • Select new vignette if necessary • Address barriers to use • Plan for use of guided imagery • Termination	• Continue self-administration of guided imagery vignette • Continue tracking intervention use and benzodiazepine use in the guided imagery practice log

### Measures

#### Anxiety and depression

The Hospital Anxiety and Depression Scale (HADS [29];) is a 14-item self-report measure of anxiety and depression symptoms for use in a medically ill patients, as it does not include the somatic symptoms of anxiety and depression that confound the assessment of distress in medically ill patients, and has demonstrated high reliability and validity in medically ill populations [30]. The measure contains seven anxiety items and seven depression items, corresponding to the two subscales. For each item, the participant is asked to identify how much a given statement is applicable (*most of the time*, *a lot of the time*, *from time to time*, *occasionally*, or *not at all*). A cut score of 8 identifies cases of anxiety and depressive disorders for each subscale, resulting in sensitivity and specificity of approximately 0.80 [30].

#### Symptom burden

The Memorial Symptom Assessment Scale-Short Form (MSAS-SF [31];) is a multidimensional symptom assessment instrument. It assesses both symptom presence and symptom distress. It assesses the occurrence of 26 physical symptoms and four psychological symptoms on a scale from 0 (“no symptom”) to 4 (“very much”). Distress is rated on a 5-point scale including not at all, a little bit, somewhat, quite a bit, and very much. The scale yields a total symptom distress score (TMSAS), a global distress index (GDI), a physical symptom distress score (PHYS), and a psychologic symptom distress score (PSYCH). In a sample of patients with cancer, Cronbach alpha was 0.80 for the GDI, 0.82 for the PHYS, 0.76 for the PSYCH, and 0.87 for the TMSAS. It also demonstrated good criterion validity in patients with cancer.

#### Health-related quality of life

The Functional Assessment of Cancer Therapy — Head and Neck (FACT-HN) version is a 27-item self-report instrument designed to assess quality of life for patients with head and neck cancer [32]. Items assess four domains: physical, social/family, emotional, and functional well-being as well as specific items assessing head and neck symptoms. The scale uses a Likert-type scale (0 to 4) to produce subscale and total scores with higher scores indicating higher quality of life. It is a reliable, valid measure of quality of life for patients with head and neck cancer [32].

#### Exploratory outcomes

Participant use of anxiolytic medications is an exploratory outcome. It will be assessed through both medical record review of prescriptions and patient reported use. All participants will record their use of any of the following medications: alprazolam, bromazepam, chlordiazepoxide, clonazepam, clorazepate, diazepam, flurazepam, and lorazepam. All participants will be given a weekly TLFB measure to track daily use of anxiolytics over the course of the study.

### Data management

Study participant research data will be collected using REDCap, a HIPAA-compliant secure web application designed to support data capture for research studies. The database is stored at the University of Colorado — Denver Development and Informatics Service Center (DISC), which is a central location for data processing and management. Individual participants and their research data will be identified by a unique study identification number. The study data entry and study management systems used by clinical sites and by the study research staff will be secured and password protected. Quality control (QC) procedures will be implemented beginning with the data entry system, and data QC checks that will be run on the database will be generated. Any missing data or data anomalies will be communicated to the site(s) for clarification/resolution. At the end of the study, all study databases will be de-identified and archived at the University of Colorado.

### Interventions

#### GIFT

The GIFT intervention consists of two in-person sessions guided by an interventionist with at least master’s level clinical training (e.g., clinical psychologist, LCSW, psychology doctoral student) and ongoing access to guided imagery exercises via MP3 player for self-administration. The intervention is guided by a study manual developed by the study PI, and reviewed by study collaborators, which details the study rationale and session content. Each interventionist will attend a training session conducted by the study PI that will provide an introduction to the study manual, a review of each intervention component, and an opportunity to participate in role-play exercises to ensure fidelity in delivering the therapy. Table 2 provides a summary of the content of the GIFT sessions.

Session 1 is held during the week of the participant’s CT simulation, usually following an orientation to RT from the clinic nurse coordinator. After explaining the rationale for treatment, the study interventionist continues to build rapport with the participant by exploring the participant’s cancer story. Using an initially unstructured approach allows the participant to highlight aspects of the cancer experience that are most relevant to him or her. The manual provides prompts about the impact of cancer on functioning that can be employed as needed.

Session 1 also includes an interactive exercise based on the biobehavioral framework of cancer stress that encourages the participant to identify his or her physical, cognitive, emotional, and behavioral manifestations of stress. Finally, the therapist provides the rationale for guided imagery as a strategy to cope with stress by focusing and directing attention and imagination. The guided imagery intervention will include interventionist-directed audio delivery of one of three guided imagery vignettes. The vignettes are sourced, with permission [33] from the University of Michigan Comprehensive Cancer Center’s Guided Imagery Library. The vignettes included in the study will be as follows: *taking a walk*, *healthy cell alliance for treatment*, and *daily intention* [34]. Each vignette is approximately 12 min. The intervention, based on established psychotherapy principles, can later be self-administered. Participants are provided with an MP3 player preloaded with audio files of each vignette. Following administration, the participant is encouraged to reflect on the impact of the guided imagery exercise on perceived stress. The end of the session focuses on planning for self-administration of the guided imagery vignette. Practice logs are provided to track self-administration.

Session 2 occurs during the first week of scheduled RT. The purpose of this session is to review and reinforce use of the intervention and to identify barriers to self-administration. This session draws from a problem-solving therapy [35] framework as patients are encouraged to plan for continued intervention use over the course of their RT.

#### Treatment as Usual (TAU)

All study participants will receive TAU which includes an orientation to RT from the clinic nurse coordinator. This will include a tour of the treatment room, as well as educational materials about RT including the process of RT and CT simulation, treatment side effects, pain management, and swallowing exercises. All study participants will be provided with an MP3 player preloaded with guided imagery audio files that they will be allowed to keep as part of study participation. Participants in the TAU group will receive it at the end of study participation.

#### GIFT intervention fidelity checks

All therapists are provided with a list of study tasks to be completed in session. We will also record study sessions and randomly select 10% of recorded sessions to review for fidelity using checklists.

#### Intervention adherence

Several strategies will be used to improve intervention and survey adherence. For participants enrolled in the intervention group, research staff will make every effort to schedule GIFT sessions at the convenience of the patient while also adhering to the protocol time constraints. GIFT sessions will be conducted on site in the Radiology Oncology Clinic, in order to provide a convenient and integrated care experience for participants. When sessions have been scheduled, the interventionist will place reminder calls to the subject the day before their scheduled session to minimize missed sessions. Research staff will also be available by telephone and email to intervention subjects on the day of sessions to provide direct assistance with any immediate barriers to attending sessions (e.g., parking difficulties, navigating to the group session location). Attendance of sessions will be monitored, recorded, and entered into an electronic database for tracking. Additionally, subject’s weekly use of the GIFT intervention outside of the session will tracked in by TLFB.

#### Anticipated risks

There are minimal risks to intervention participants. However, participants may experience distress as they are asked to reflect on aspects of their cancer experience during the GIFT intervention and the on the surveys, which may cause distress. If this distress is acute, psychosocial support will be available. Should study PI believe at any point in the study that participation is detrimental to the participant’s health, the subject’s participation will be terminated, and the subject will be referred to other relevant treatment resources as appropriate (i.e., mental health resources). Participants also have the right to voluntarily withdraw from the study at any time; if this occurs, research staff will verify their decision and obtain details on their reason(s) for withdrawal.

There are no plans for ancillary or posttrial care, as this trial is testing a psychological intervention that confers very low risks of either short- or long-term harm. In the unlikely event that subjects are injured as a result of procedures associated with this study, they are advised to seek appropriate medical care immediately and to inform the principal investigator as soon as possible. Participants are provided a detailed explanation of this policy during the informed consent process.

#### Concomitant care

All prescription medications related to the study aims taken during study participation will be recorded in the study database. For this protocol, relevant prescription medications include anxiolytic medication (i.e., alprazolam, bromazepam, chlordiazepoxide, clonazepam, clorazepate, diazepam, flurazepam, and lorazepam). These medications will be identified through chart review or self-report. Given the potential for distress and need for support in this population, it would be unethical to limit participants use of additional resources to manage their distress; therefore, participants in both randomization group are free to utilize any other resources, such as additional psychotherapy support or stress management interventions.

### Study and data safety monitoring

The principal investigator will be responsible for the conduct of this study, overseeing participant safety, executing the data and safety monitoring (DSM) plan, and complying with all reporting requirements to local and federal authorities. This oversight will be accomplished through additional oversight from the Data and Safety Monitoring Committee (DSMC) at the University of Colorado Cancer Center (CU Cancer Center). Per the CU Cancer Center Institutional DSM Plan, UAPs and reportable AEs are reported to the DSMC, COMIRB, and the sponsor per study protocol. All UAPs and reportable AEs are to be reported to the DSMC within 5 business days of the sponsor investigator receiving notification of the occurrence. Study audits conducted by the DSMC will consist of a review of the regulatory documents, consent forms, and source data verification. Documentation of the audit conducted by the DSMC will then need to be submitted to the IRB of record at the time of the IRB’s continuing review of this trial.

The nature of this trial is low risk, and we do not anticipate any nonserious adverse events; however, in the event that one occurs, the sponsor-investigator must record nonserious adverse events and report to DSMC and COMIRB according to timetable for reporting specified in the Data Safety Monitoring Plan and per COMIRB reporting requirements. Reporting will be done by the Oncology Clinical Research Support Team (OCRST) and principal investigator.

This study will follow COMIRB’s guidance for unanticipated problems reporting and the DSMC’s requirements. Adverse events, noncompliance, and protocol violations will be recorded and reported as required either promptly (within 5 days of sponsor-investigator’s knowledge) or at the time of the study’s continuing review. It is the responsibility of the PI to report incidents or events that meet the criteria for unanticipated problem reporting to COMIRB using their standard unanticipated problem form. In the event of any major modifications to the study protocol, the changes will be immediately communicated to all relevant parties, including study participants, COMIRB, DSMC, OCRST, the study sponsor, and all research staff and co-investigators.

### Sample size

We expect that we will be able to enroll 72 people with measured anxiety who will be randomized into either TAU only (*n* = 36) or the GIFT intervention group (*n* = 36). This number was calculated based on planned analyses for the secondary outcome related to changes in anxiety and depression as measured by the HADS. It is also sufficient for evaluating the primary feasibility and acceptability aims of the project. We use the values for means and standard deviations from a study of the HADS in patients with HNC receiving RT [19]. Significance levels (alpha) are set at 0.05 for a two-sided independent samples equal-variance *t*-test. For the HADS-A, the mean score in controls at the end of RT was 6.9 (*SD* = 5.0). With group sample sizes of 36, we will have 80% power to reject the null hypothesis of equal means when the HADS-A score in the guided imagery group is ≤ 3.6 (a ≥ 48% difference between the scores for each group at the end of RT). For the HADS-D, the mean score in controls at the end of RT was 11.2 (*SD* = 5.5). Group sample sizes of 36 and 36 achieve 80% power to reject the null hypothesis of equal means when HADS-D score in the guided imagery group is ≤ 7.5 (a ≥ 33% difference between the scores at the end of RT).

### Planned data analysis

The primary study aim is to examine the feasibility and acceptability of the GIFT intervention. Therefore, the statistical analyses used to evaluate this aim will primarily be descriptive and based on qualitative analyses of semi-structured interview data. However, secondary and exploratory analyses will focus on parameters that will also be important for conducting a future trial.

The recruitment, enrollment, and retention processes will determine feasibility. This includes the number of patients eligible and approached for participation, number enrolled in the study, and number who completed study procedures, including intervention sessions and study measures. Planned statistical analyses include frequencies, rations of patients who enroll versus do not enroll in the study, and percentages of participants who complete study measures. This will include analysis of level of missing data. Prevalence of missing data will inform appropriate methods to account for missingness.

Acceptability will be evaluated through the qualitative interviews. These interviews will assess the acceptability, feasibility, and usefulness of the GIFT intervention. Analysis will begin with the transcription of each semi-structured interview into the coding software program. Qualitative analyses will be conducted using Atlas.ti software, which will store, code, and categorize data transcripts. Data will be analyzed with a constant comparative approach [28] — an inductive approach to data analysis through which each piece of data (e.g., statements, emerging themes) is compared to other pieces of data and evaluated for similarities and/or differences. In qualitative research, it is generally accepted that data collection continues until “saturation” had been met. Saturation occurs once a researcher has collected enough case data such that data provided by additional cases does not provide new information or themes. It has been suggested, from studies that utilize individualized interviews to develop and understand nuances of theory, that between 12 and 30 participants are typically needed to reach saturation [27]. Acceptability analyses will also include percentage of participants who were in the GIFT intervention group who reported self-administration of the guided imagery vignettes outside of session as assessed by TLFB.

We will conduct independent samples *t*-tests to test the secondary aims to support preliminary efficacy of the GIFT intervention to reduce anxiety and depression. Data will be analyzed using repeated measures analyses or mixed models to study changes over time in HADS scores within treatment groups and to test for interactions between time and treatment groups. Similar methods will be used to evaluate health-related quality of life and symptom burden. The datasets used and/or analyzed during the current study will be available from the corresponding author upon reasonable request. Findings from this study will be written for publication and submitted to health psychology/psychosocial oncology journals and conferences. They will also be shared on relevant Colorado behavioral oncology research online platforms including our new Connecting Colorado-Behavioral Oncology website.

## Discussion

This is a novel clinical intervention study with the potential for wide-ranging clinical impact. HNC has been labeled the “most psychologically traumatic cancer to experience.” ( [7] p 2). Depression and anxiety are highly prevalent in this population and can be exacerbated by cancer-directed treatment. In fact, symptoms of depression affect immunocompetence, treatment adherence, and other aspects of health-related quality of life that persist after the treatment completion [7]. Patients with anxiety report a greater impact of their disease including intrusive thoughts and avoidance behaviors [19]. Yet, there is a paucity of well-designed randomized controlled trials targeting psychological distress that have been evaluated for feasibility and acceptability in patients with HNC receiving RT in an outpatient setting. However, there is evidence that patients with HNC do respond well to brief interventions targeting psychological distress, and thus, establishing effective interventions is critically important [36, 37]. This project aims to meet this patient need.

The GIFT intervention relies on empirically supported principles and practices, offering promise to reduce distress in a psychologically vulnerable medical population. Patients with HNC are underserved with regard to behavioral medicine interventions. There are no published interventions of GI in patients with HNC despite its efficacy [35] in other populations of patients with cancer. In addition to being easily integrated into patients’ treatment schedules, the GIFT intervention was designed to be brief, allowing for the quick acquisition of skills. These sessions also aim to increase participant awareness of the biopsychosocial manifestations of stress. The participant is provided with the tools and support to self-administer the skills learned in the intervention session as needed over the course of his or her treatment. This maximizes time with mental health professionals, a scarce resource in many treatment settings. By promoting self-management of psychological distress, participants will feel more equipped to handle a physically, mentally, and emotionally demanding treatment.

The innovation in this project is twofold. Primarily, the project evaluates the feasibility and acceptability of a guided imagery intervention that has the potential to reduce psychological distress in a vulnerable population. The intervention was designed to be brief and evaluate feasibility of intervention integration into a radiation oncology clinic, demonstrating the translational nature of the project. The secondary aim will provide preliminary data needed to inform the design of larger efficacy trials. The exploratory aim of monitoring anxiolytic medication use in this population is a novel area of study. These medications are often used to address treatment-related anxiety symptoms in patients with cancer [38] but can produce side effects including physical and psychological dependence [39, 40]. Our exploratory analyses will allow us to understand more about the possible role of our behavioral intervention impacting the need for anxiolytic use in this population.

The successful implementation of this interdisciplinary research holds the potential to lower psychological distress and improved health-related quality of life. This is particularly significant in a population of patients who are highly vulnerable to anxiety and depression as they undergo onerous treatment and cope with heavy symptom burden. These psychological symptoms can influence treatment adherence and survival; thus, behavioral intervention is paramount. Guided imagery holds the potential to significantly improve distressing psychological symptoms in vulnerable patients facing intensive treatment and heavy symptom burden. This intervention will directly address psychological distress to establish preliminary efficacy that will lay the groundwork for larger efficacy trials.

## Supplementary Information

**Additional file 1.**

## Data Availability

Not applicable.

## Abbreviations

AEs: Adverse events
ACS: American Cancer Society
DISC: Denver Development and Informatics Service Center
DSMC: Data and Safety Monitoring Committee
FACT-HN: Functional Assessment of Cancer Therapy — Head and Neck version
GI: Guided imagery
GIFT: Guided imagery intervention
HADS-A: Hospital Anxiety and Depression Scale — Anxiety subscale
HADS-D: Hospital Anxiety and Depression Scale — Depression subscale
HNC: Head and neck cancer
IIT: Investigator-initiated trial
MSAS-SF: Memorial Symptom Assessment Scale-Short Form
OCRST: Oncology Clinical Research Support Team
QC: Quality control
RCT: Randomized control trial
RT: Radiotherapy
TLFB: Timeline follow-back
TLFB-I: Timeline follow-back for intervention use
TLFB-A: Timeline follow-back for anxiolytic use
UAPs: Unanticipated problems
UCCC: University of Colorado Cancer Center

## References

[1] Jemal A, Thomas A, Murray T, Thun M (2002). Cancer statistics, 2002. CA Cancer J Clin.

[2] Vokes EE, Weichselbaum RR, Lippman SM, Hong WK (1993). Medical progress - head and neck-cancer. N Engl J Med.

[3] Ringash J (2015). Survivorship and quality of life in head and neck cancer. J Clin Oncol.

[4] Loorents V, Rosell J, Willner HS, Borjeson S (2016). Health-related quality of life up to 1 year after radiotherapy in patients with head and neck cancer (HNC). Springerplus..

[5] Frampton M (2001). Psychological distress in patients with head and neck cancer: review. Br J Oral Maxillofac Surg.

[6] Silverman S (1999). Oral cancer - complications of therapy. Oral Surg Oral Med Oral Pathol Oral Radiol Endod.

[7] Howren MB, Christensen AJ, Karnell LH, Funk GF (2013). Psychological factors associated with head and neck cancer treatment and survivorship: evidence and opportunities for behavioral medicine. J Consult Clin Psychol.

[8] Badr H, Gupta V, Sikora A, Posner M (2014). Psychological distress in patients and caregivers over the course of radiotherapy for head and neck cancer. Oral Oncol.

[9] Gritz ER, Carmack CL, de Moor C, Coscarelli A, Schacherer CW, Meyers EG (1999). First year after head and neck cancer: quality of life. J Clin Oncol.

[10] Breitbart W, Holland J (1988). Psychosocial-aspects of head and neck-cancer. Semin Oncol.

[11] Frick E, Tyroller M, Panzer M (2007). Anxiety, depression and quality of life of cancer patients undergoing radiation therapy: a cross-sectional study in a community hospital outpatient centre. Eur J Cancer Care (Engl).

[12] Semple CJ, Dunwoody L, Kernohan WG, McCaughan E, Sullivan K (2008). Changes and challenges to patients’ lifestyle patterns following treatment for head and neck cancer. J Adv Nurs.

[13] Ichikura K, Yamashita A, Sugimoto T, Kishimoto S, Matsushima E (2016). Persistence of psychological distress and correlated factors among patients with head and neck cancer (vol 14, pg 42, 2016). Palliat Support Care.

[14] Zeller JL (2006). High suicide risk found for patients with head and neck cancer. JAMA.

[15] Parkin DM, Bray F, Ferlay J, Pisani P (2005). Global cancer statistics, 2002. CA Cancer J Clin.

[16] Duffy SA, Ronis DL, Valenstein M, Lambert MT, Fowler KE, Gregory L (2006). A tailored smoking, alcohol, and depression intervention for head and neck cancer patients. Cancer Epidemiol Biomar.

[17] Ojo B, Genden EM, Teng MS, Milbury K, Misiukiewicz KJ, Badr H (2012). A systematic review of head and neck cancer quality of life assessment instruments. Oral Oncol.

[18] Chen AM, Hsu S, Felix C, Garst J, Yoshizaki T. Effect of psychosocial distress on outcome for head and neck cancer patients undergoing radiation. Laryngoscope. 2017;128(3):641-645. 10.1002/lary.26751 28714543

[19] Chen AM, Daly ME, Farwell DG, Vazquez E, Courquin J, Lau DH (2014). Quality of life among long-term survivors of head and neck cancer treated by intensity-modulated radiotherapy. JAMA Otolaryngol.

[20] Rossetti A, Chadha M, Lucido D, Hylton D, Loewy J, Harrison L (2014). The impact of music therapy on anxiety and distress in patients undergoing simulation for radiation therapy (RT). Int J Radiat Oncol.

[21] Neilson K, Pollard A, Boonzaier A, Corry J, Castle D, Smith D (2013). A longitudinal study of distress (depression and anxiety) up to 18 months after radiotherapy for head and neck cancer. Psychooncology..

[22] Neilson KA, Pollard AC, Boonzaier AM, Corry J, Castle DJ, Mead KR (2010). Psychological distress (depression and anxiety) in people with head and neck cancers. Med J Aust.

[23] Wu YS, Lin PY, Chien CY, Fang FM, Chiu NM, Hung CF (2016). Anxiety and depression in patients with head and neck cancer: 6-month follow-up study. Neuropsychiatr Dis Treat.

[24] Kelly C, Paleri V, Downs C, Shah R (2007). Deterioration in quality of life and depressive symptoms during radiation therapy for head and neck cancer. Otolaryngol Head Neck Surg.

[25] Roffe L, Schmidt K, Ernst E (2005). A systematic review of guided imagery as an adjuvant cancer therapy. Psychooncology.

[26] Kolcaba K, Fox C (1999). The effects of guided imagery on comfort of women with early stage breast cancer undergoing radiation therapy. Oncol Nurs Forum.

[27] Guest G, Bunce A, Johnson L (2006). How many interviews are enough? An experiment with data saturation and variability. Field Method.

[28] Boeije H (2002). A purposeful approach to the constant comparative method in the analysis of qualitative interviews. Qual Quant.

[29] Zigmond AS, Snaith RP (1983). The Hospital Anxiety and Depression Scale. Acta Psychiatr Scand.

[30] Bjelland I, Dahl AA, Haug TT, Neckelmann D (2002). The validity of the Hospital Anxiety and Depression Scale. An updated literature review. J Psychosom Res.

[31] Chang VT, Hwang SS, Feuerman M, Kasimis BS, Thaler HT (2000). The Memorial Symptom Assessment Scale-Short Form (MSAS-SF). Cancer..

[32] List MA, D'Antonio LL, Cella DF, Siston A, Mumby P, Haraf D (1996). The performance status scale for head and neck cancer patients and the functional assessment of cancer therapy-head and neck scale. A study of utility and validity. Cancer..

[33] Casselman C (2017). Permission to use guided imagery scripts. In: PhD EK, editor.

[34] Center UoMCC (2017). Guided Imagery Library.

[35] Andersen BL, Yang HC, Farrar WB, Golden-Kreutz DM, Emery CF, Thornton LM (2008). Psychologic intervention improves survival for breast cancer patients: a randomized clinical trial. Cancer..

[36] Katz MR, Irish JC, Devins GM (2004). Development and pilot testing of a psychoeducational intervention for oral cancer patients. Psychooncology..

[37] Head BA, Keeney C, Studts JL, Khayat M, Bumpous J, Pfeifer M (2011). Feasibility and acceptance of a telehealth intervention to promote symptom management during treatment for head and neck cancer. J Support Oncol.

[38] Triozzi PL, Goldstein D, Laszlo J (1988). Contributions of benzodiazepines to cancer therapy. Cancer Investig.

[39] Soyka M (2017). Treatment of benzodiazepine dependence. N Engl J Med.

[40] Day GL, Blot WJ, Shore RE, McLaughlin JK, Austin DF, Greenberg RS (1994). Second cancers following oral and pharyngeal cancers: role of tobacco and alcohol. J Natl Cancer Inst.

