# Supplementary material for: Guided imagery for treatment (GIFT): protocol of a pilot trial of guided imagery versus treatment as usual to address radiotherapy-related distress in head and neck cancer
Source: Pilot Feasibility Stud. 2022 Sep 5;8:199. doi: 10.1186/s40814-022-01134-9 (PMC9446833; doi:10.1186/s40814-022-01134-9)
Supplement: Supplementary file 1 — Additional file 1. [file 40814_2022_1134_MOESM1_ESM.docx]

Additional file

**Consent and Authorization Form**

**Principal Investigator: Elissa Kolva PhD**

**COMIRB No: 18-1100**

**Version Date: April 10, 2020**

**Study Title:** *Effect of guided imagery for radiotherapy-related distress: A randomized*

*controlled trial for patients with head and neck cancer.*

You are being asked to be in a research study. This form provides you with information about the study. A member of the research team will describe this study to you and answer all of your questions. Please read the information below and ask questions about anything you don’t understand before deciding whether or not to take part.

**Why is this study being done?**

The goal of this study is to evaluate the feasibility of conducting a guided imagery (GI) intervention for symptoms of anxiety and depression in patients with head and neck cancer (HNC) compared to treatment as usual.

You are being asked to be in this research study because you have been identified as a patient starting radiotherapy (RT) for an HNC diagnosis. Guided imagery is a behavioral relaxation technique involving the visualization of calming images and is considered an adjuvant cancer therapy.

**Other people in this study**

Up to 100 people from your area will participate in the study.

**What happens if I join this study?**

If you join the study, you will be asked to sign this consent form. You will be given a copy to keep and the original form will be kept at the clinic. You can withdraw from the study at any time and without giving a reason. Participating in this study will not affect the standard medical care you receive. You will be “randomized” into one of two study groups described below. Randomization means that you are put into a group by chance. Neither you nor the study staff can choose the group you will be in. You have an equal chance of being placed in either group.

The first group will receive treatment as usual, which includes education about treatment and access to psychosocial support available through the UC Cancer Center. The second group will also receive the treatment as usual described above. In addition, you will also receive two in person, individual guided imagery sessions (one t the time of your CT simulation, and one during your first week of RT) and be asked to self administer GI throughout your treatment. The next section of this form lists what will be expected of you if you join this study.

**Study Procedures**

**Informed Consent**

This informed consent form will be discussed with you and you will be given a copy of this document.

**Demographics, Medical and Cancer History**

Before you start the study we will record your date of birth, race, ethnicity and other demographic characteristics. We will also record your and complete medical history. This history will include the background and progress of your cancer and any treatments you have received for your disease, and other relevant medical characteristics.

**Review of Current Medications**

You may continue taking all prescribed or over the counter medications as directed. Starting with the screening/baseline visit, we will record the medications you are taking during the study.

**Questionnaires**

You will be asked to complete questionnaires throughout the course of this study, which

will ask about how RT treatment is affecting your quality of life. The questionnaires will assess your mood, health-related quality of life, current symptoms and use of anxiety medication and if appropriate, use of the guided imagery intervention. The questionnaires should not take more than ten minutes to complete each week. They will also ask about your use of the services provided by UC Cancer Center and what type of impact they are having on your quality of life.

**Guided Imagery**

The GI consists of two individual, personalized sessions that will introduce a relaxation practice prior to CT simulation and during the first week of RT. These two sessions will be recorded to ensure accuracy. You will be able to choose from three types of relaxation practices, which will be explained prior to your first session. You will then be asked to track weekly use of the guided imagery intervention during RT.

**Treatment as Usual**

The treatment as usual condition involves education about treatment and access to psychosocial support available through UC Cancer Center. These services are available for every patient throughout the course of their treatment.

**Follow-Up Interview**

If you are in the guided imagery group, you will be asked to have a follow-up interview. This visit will occur after you complete your Week 12 study visit. It can be completed in-person or over the phone, and will include speaking with study staff about the use of the

oncology resource services and GI (if applicable). This interview will be recorded so that it can be transcribed and analyzed appropriately.

**Study Visits**

Please refer to the table below for the schedule of study visit procedures.


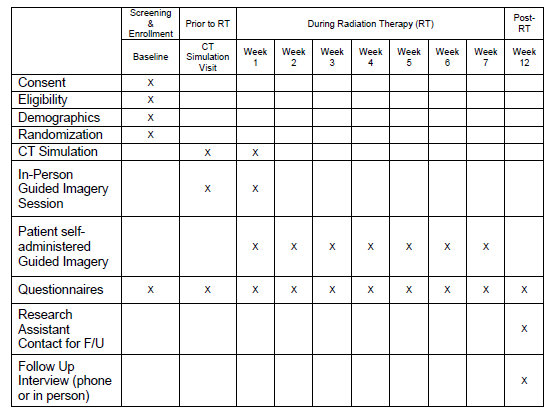


**How Long Will I be in the Study?**

Study participation will last approximately four months. All sessions will be completed in

approximately 12 to 16 weeks after initiation RT, depending on the group you are randomized to, treatment as usual or guided imagery.

**What are the possible discomforts or risks?**

While in this study you may experience discomfort when asked questions about anxiety

symptoms, depression, and basic demographics. Please feel free to skip any questions

that make you feel uncomfortable. If you have any mental concerns that you feel need to be discussed with a mental health professional while you are participating in this trial call Dr. Elissa Kolva, her office phone number is 720-848-8090. Other possible risks include loss of confidentiality. There is a risk that people outside of the research team will see your research information. We will do all that we can to protect your information, but it cannot be guaranteed. To help prevent the loss of confidentiality, no names or medical record numbers will be used on the questionnaire or in any study database. Study staff will assign a research identification number to each participant that will be used on the questionnaire. Only Dr. Kolva and the study staff will have access to the key linking research identification numbers to participants’ names. This key will be kept in a secure electronic file that is password protected.

**What are the possible benefits of the study?**

You may or may not benefit for participation. The goal of this intervention is to create an

intervention that can be used in the future for patients with head and neck cancer. However, there is no guarantee that your health will improve if you join this study. Also,

there could be risks to being in this study. If there are risks, these are described in the

section describing the discomforts or risks.

**Are there alternative treatments?**

There may be other ways of treating your symptoms of psychosocial distress. These other ways include seeking relief through your primary health or mental health provider. There are medications to alleviate symptoms or you could also choose to get no treatment at all. You should talk to your doctor about your choices. Make sure you understand all of your choices before you decide to take part in this study. You may leave this study and still have these other choices available to you.

**Will I be paid for being in the study?**

You will not be paid to be in the study. However all participants will receive an mp3 player that they can keep following study completion. Participants in the guided imagery group may be eligible to participate in a qualitative interview. Those participants who complete the qualitative interview will receive a $25 gift card.

**Will I have to pay for anything?**

It will not cost you anything to be in the study.

**Is my participation voluntary?**

Taking part in this study is voluntary. You have the right to choose not to take part in this study. If you choose to take part, you have the right to stop at any time. If you refuse or decide to withdraw later, you will not lose any benefits or rights to which you are entitled. If there are any new findings during the study that may affect whether you want to continue to take part, you will be told about them.

**Can I be removed from this study?**

The study doctor may decide to stop your participation without your permission if the study doctor thinks that being in the study may cause you harm, or for any other reason.

**What happens if I am injured or hurt during the study?**

If you have an injury while you are in this study, you should call Dr. Elissa Kolva

immediately. Her phone number is 720-848-8090*.* We will arrange to get you medical care if you have an injury directly caused by this research. However, you or your insurance company will have to pay for that care.

**Whom do I call if I have questions?**

The researcher carrying out this study is Dr. Kolva. You may ask any questions you have now. If you have questions, concerns, or complaints later, you may call Dr. Kolva at 720-848-8090. You will be given a copy of this form to keep. You may have questions about your rights as someone in this study. You can call Dr. Kolva with questions. You can also call the responsible Institutional Review Board (COMIRB). You can call them at 303-724-1055. A description of this clinical trial will be available on http://www.Clinical Trials.gov, as required by U.S. Law. This Web site will not include information that can identify you. At most, the Web site will include a summary of the results. You can search this Web site at any time.

**Who will see my research information?**

The University of Colorado Denver (UCD) and its affiliated hospital(s) have rules to protect information about you. Federal and state laws including the Health Insurance Portability and Accountability Act (HIPAA) also protect your privacy. This part of the consent form tells you what information about you may be collected in this study and who might see or use it. The institutions involved in this study include

- University of Colorado Denver

- University of Colorado Hospital

We cannot do this study without your permission to see, use and give out your information. You do not have to give us this permission. If you do not, then you may not join this study. We will see, use and disclose your information only as described in this form and in our Notice of Privacy Practices; however, people outside the UCD and its affiliate hospitals may not be covered by this obligation. We will do everything we can to maintain the confidentiality of your personal information but confidentiality cannot be guaranteed. The use and disclosure of your information has no time limit. You can cancel your permission to use and disclose your information at any time by writing to the study’s Principal Investigator (PI), at the name and address listed below. If you do cancel your permission to use and disclose your information, your part in this study will end and no further information about you will be collected. Your cancellation would not affect information already collected in this study.

Dr. Elissa Kolva

University of Colorado

Department of Oncology

12605 E 16th Ave

Aurora, CO 80045

720-848-8090

Both the research records that identify you and the consent form signed by you may be

looked at by others who have a legal right to see that information, such as:

 People at the Colorado Multiple Institutional Review Board (COMIRB)

 The study doctor and the rest of the study team.

 Officials at the institution where the research is conducted and officials at other

institutions involved in this study who are in charge of making sure that we follow all

of the rules for research We might talk about this research study at meetings. We might also print the results of this research study in relevant journals. However, we will always keep the names of the research subjects, like you, private.

 Some things we cannot keep private. If you give us any information about child

abuse or neglect we have to report that to Colorado Social Services. Also, if we

get a court order to turn over your study records, we will have to do that.

 In addition*,* if you tell us you are going to physically hurt yourself or someone

else, we have to report that to the Colorado State Police. Also, if we get a court

order to turn over your study records, we will have to do that.

You have the right to request access to your personal health information from the

Investigator.

**Information about you that will be seen, collected, used and disclosed in this study:**

-Name and Demographic Information (age, sex, ethnicity, address, phone number,

etc.

- Portions of your previous and current Medical Records that are relevant to this

study, including but not limited to Diagnosis(es), History and Physical.

- Psychological and mental health tests

- Alcoholism, Alcohol or Drug abuse

We respect your right to privacy. But there are some things we cannot keep private.

If you give us information about child neglect or child abuse, we have to report that to

Social Services. If you give us information about someone hurting someone else, we

have to report that to the police. If a court orders us to hand over your study records,

we have to hand them over to the court.

**What happens to Data that is collected in this study?**

Scientists at the University of Colorado Denver and the hospitals involved in this study

work to find the causes and cures of disease. The data collected from you during this

study are important to this study and to future research. If you join this study:

- The data given by you to the investigators for this research and so no longer

belong to you.

- Both the investigators and any sponsor of this research may study your data

collected from you.

- If data is in a form that identifies you, UCD or the hospitals involved in this

study may use them for future research only with your consent or IRB approval.

-Any product or idea created by the researchers working on this study will not

belong to you.

- There is no plan for you to receive any financial benefit from the creation, use

or sale of such a product or idea.

**Agreement to be in this study and use my data**

The research project and the procedures associated with it have been explained to me.

The experimental procedures have been identified and no guarantee has been given about the possible results. I will receive a signed copy of this consent form for my records. I agree to participate in this study. My participation is voluntary and I do not have to sign this form if I do not want to be part of this research study.

Signature: Date:

Print Name:

Consent form explained by: Date:

Print Name:
